# Chemical Composition of Volatile and Extractive Components of Canary (Tenerife) Propolis

**DOI:** 10.3390/molecules29081863

**Published:** 2024-04-19

**Authors:** Valery A. Isidorov, Andrea M. Dallagnol, Adam Zalewski

**Affiliations:** 1 Institute of Forest Sciences, Białystok University of Technology, 15-351 Białystok, Poland; 2 Instituto de Materiales de Misiones (CONICET-UNaM), Felix de Azara 1552, Posadas 3300, Argentina; andymicd@gmail.com; 3 Department of Experimental Physiology and Pathophysiology, Medical University of Białystok, 15-222 Bialystok, Poland; adam90z@o2.pl

**Keywords:** propolis, chemical composition, volatile compounds, extractive components, terpenoids, resorcinol derivatives, lignans

## Abstract

The vegetation of the Canary Islands is characterized by a large number of endemic species confined to different altitudinal levels. It can be assumed that these circumstances determine the characteristic features of the chemical composition of local beekeeping products, including propolis. We report, for the first time, the chemical composition of propolis from Tenerife (Canary Islands). The volatile emissions of three propolis samples collected from different apiaries are represented by 162 C_1_–C_20_ compounds, of which 144 were identified using the HS-SPME/GC-MS technique. The main group of volatiles, consisting of 72 compounds, is formed by terpenoids, which account for 42–68% of the total ion current (TIC) of the chromatograms. The next most numerous groups are formed by C_6_–C_17_ alkanes and alkenes (6–32% TIC) and aliphatic C_3_–C_11_ carbonyl compounds (7–20% TIC). The volatile emissions also contain C_1_–C_6_ aliphatic acids and C_2_–C_8_ alcohols, as well as their esters. Peaks of 138 organic C_3_–C_34_ compounds were recorded in the chromatograms of the ether extracts of the studied propolis. Terpene compounds form the most numerous group, but their number and content in different samples is within very wide limits (9–63% TIC), which is probably due to the origin of the samples from apiaries located at different altitudes. A peculiarity of the chemical composition of the extractive substances is the almost complete absence of phenylcarboxylic acids and flavonoids, characteristic of *Apis mellifera* propolis from different regions of Eurasia and North America. Aromatic compounds of propolis from Tenerife are represented by a group of nine isomeric furofuranoid lignans, as well as alkyl- and alkenyl-substituted derivatives of salicylic acid and resorcinol.

## 1. Introduction

Propolis is one of the most valuable beekeeping products, but a honeybee colony’s need for it is not very great. Therefore, only a small proportion of worker bees are involved in collecting raw plant materials for its production. Meanwhile, the role of propolis in ensuring the survival of the colony in a far-from-sterile environment cannot be overestimated: it has high activity against various pathogens [1,2,3] and plays a key role in the ‘social immunity’ of bees [4,5].

In recent years, increasing attention has been paid to propolis as an antidote to human microbial pathogens. In terms of antisepticity, propolis stands out among most other beekeeping products, second only to bee venom in terms of potency [6,7,8]. It exhibits a wide range of medicinal properties, which are not limited to antimicrobial action, as reflected in a number of recent comprehensive reviews [9,10,11,12]. In particular, its beneficial effects on the human digestive system, in the treatment of a number of gynecological diseases, and in the healing of wounds and burns, as well as in dermatology and a number of oncological diseases, have been shown [13,14,15,16,17].

It is generally accepted that the antibacterial activity and other medicinal properties of propolis are due primarily to phenolic compounds of plant origin, flavonoids, phenolcarboxylic acids and their esters [9,10,18]. These natural compounds are found not only in plant tissues but also in their external secretions in the form of resin, exudates and leakage from wounded tissues, and perform protective functions against pathogens and parasites. It is these properties that encourage bees to collect these materials, in need of a means of combating pests and pathogens in their hives. Bees are selective and preferential in relation to plant sources of raw materials for the production of propolis [2,19,20,21], and when searching for them, they are guided by various insufficiently studied olfactory and optical signals. A consequence of the selectivity of bees that deliver raw materials is the existence of certain regional ‘types’ or varieties of propolis [22]. For example, the middle latitudes of Eurasia and North America are characterized by the ‘poplar type’, the plant precursor of which is exudates on the buds of different types of poplar containing the flavonoid aglycone, phenylcarboxylic acids and their derivatives [18,19,23]. In boreal regions, outside the growing range of poplar, bees collect resin from the buds of downy birch (*Betula pubescens*), which are not only rich in flavonoids but also contain large amounts of sesquiterpenol esters of phenylpropenoid acids [23,24]. Another source of resin are aspen (*Populus tremula* and *P. tremuloides*) buds, characterized by a high content of phenylpropenoid glycerides [23,25,26]. In the boreal zone, a mixed ‘birch-aspen type’ of propolis is often found [23], and on the border with the mid-latitude zone, poplar markers are also found in its composition [27]. In other phytogeographical zones and regions, there are local sources of resin acceptable to foraging bees, and there they produce specific types of propolis, such as green Brazilian propolis from the tropical zone [28] or Argentine Andean propolis [29]. The botanical predecessor of the former is *Baccharis dracunculifolia*, and that of the latter is *Larrea nitida*.

In isolated island areas, often rich in endemic plant species, one can also expect the existence of propolis with a specific chemical composition that allows it to be considered a separate variety. This applies to diterpenoids-rich propolis from the Greek islands [30] and to Pacific propolis from Okinawa and Taiwan [31]. Such isolated areas include the islands of the Canary archipelago, where beekeeping has long been practiced, but its products still remain poorly understood. In particular, there is only one report on the chemical composition of two propolis samples from the island of Gran Canaria [32]. Judging by the data presented, these samples demonstrate significant qualitative differences both from propolis from the temperate zone and from its known varieties of tropical origin. This is undoubtedly due to the presence on the island of specific plant sources of resins or other types of secretions that are attractive to the bees bred on it. It would be interesting to study the chemical composition of propolis from other Canary Islands, since each of them is characterized by biogeographical differences. This work reports for the first time the chemical composition of propolis from Tenerife, the largest of the islands of the archipelago and possessing plant species endemic only to this island [33].

## 2. Results

### 2.1. Composition of Volatile Components of Propolis

Determination of the volatile organic components (VOCs) of propolis was carried out using the method of solid phase microextraction combined with gas chromatography with mass spectrometric detection (HS-SPME/GC-MS). Chromatograms recorded using this method contained peaks of 162 C_1_–C_20_ compounds belonging to different classes of organic substances. Table 1 shows the composition of VOCs, grouped by class of organic compounds, as well as the substances included in each group in order of increasing retention index. The largest contribution to the total ion current (TIC) of the chromatograms was made by the group of terpenoids, numbering 72 compounds. The monoterpenoid subgroup, formed by 34 individual components (11 of them were found in all three samples), accounted for 27–56% of the TIC. However, 12 monoterpenoids were recorded in only one of the three samples. An even greater difference was observed in the subgroup of sesquiterpenoids: 27 out of a total of 36 were found in only one of the samples.

The second largest group of VOCs was formed by C_6_–C_17_ alkanes and alkenes, the contribution of which to the TIC chromatograms varied greatly and ranged from 6% to 32%. It is interesting that even-numbered homologs predominated in terms of their contribution to the TIC. The VOCs contained 17 aliphatic carbonyl compounds, the proportion of which ranged from 7% to 20% of the TIC. The largest amounts were acetone, nonanal and heptanal. In addition, the fugitive emissions contained C_2_–C_8_ aliphatic alcohols and C_1_–C_6_ acids, as well as seven esters. However, the latter were found in only one of the samples (Pr-1). Of the seven aromatic compounds, toluene and *p*-cymene were present in the highest amounts in all three samples.

### 2.2. Extractive Components

Chromatograms of ether extracts of all three propolis samples were formed by peaks of 138 organic compounds, most of which contained polar groups and were recorded in the form of TMS derivatives. The identified compounds could be divided into 12 groups, as shown in Table 2. As in the case of VOCs, the largest group was formed by terpenoids, but its structure was different. It was formed by 10 sesquiterpenes, 22 diterpenes and 13 triterpenes. Sesquiterpenes were present only in small quantities (1.2–1.7% TIC) and only in extracts from two propolis samples. Without exception, all diterpenes were classified as resin acids and related C_20_ compounds (totarol and neoabietal). Three compounds with retention indices of 2515, 2665 and 2596 were conditionally assigned to this group based on the presence of characteristic ions in the mass spectra and the MS pattern of ion fragmentation. A subgroup of triterpenoids was represented by tetracyclic lanosterol, dihydrolanosterol and masticadienoic acid, as well as pentacyclic compounds of the oleanane, ursane and lupane group. The largest amount (21.4% TIC) and the largest number of triterpenes were found in the extract from sample 1.

The second largest group was formed by 16 C_17_–C_33_ *n*-alkanes and 10 C_23_–C_33_ alkenes; the contribution of this group to the TIC was significant and amounted to 15–48%. The group of aliphatic acids (11–21% TIC) included 21 compounds with a number of carbon atoms from 9 (azelaic acid) to 34. Aliphatic compounds also included relatively small amounts of normal C_18_–C_34_ alcohols and aldehydes, and these were not detected in one of the samples (Pr-2).

The group of aromatic compounds included four substituted alkyl and alkenyl resorcinols, five alkyl and alkenyl salicylates and nine furofuranoid lignans. If representatives of the last subgroup were present in the extracts of all three propolis samples, then resorcinol derivatives were found in two of them and substituted salicylates only in one.

The contribution of compounds not assigned to any of the 12 groups amounted to 0.1–5.5% TIC, and the share of unidentified components accounted for 3.5–10.5% TIC.

## 3. Discussion

Until now, there has been only one report on the chemical composition of Canarian (Gran Canaria) propolis [32]. Although Tenerife and Gran Canaria are located close to each other, the composition of their vegetation cover is markedly different: they have many endemic species that are found only on one of them [33]. Therefore, it seems interesting to compare the chemical composition of propolis originating from these islands.

The data obtained on the composition of propolis from Tenerife and Gran Canaria differ in many aspects, and in some cases, this may be explained by differences in the experimental technique. For example, the list of VOCs shown in Table 1 determined by the HS-SPME/GC-MS method includes a much larger number of compounds than those isolated from propolis by hydrodistillation, which loses the most volatile compounds. The list of extracted compounds (Table 2) does not contain sugars and related sugar acids and alcohols, while in the work [32], their share in one propolis sample accounts for approximately 56% of TIC, and in the second, approximately 10% of TIC. This is due to the fact that the weakly polar diethyl ether we used for extraction dissolves practically no polar sugars, unlike 70% aqueous ethanol.

Another difference is that the authors of the cited work note that the chemical composition of the extracts from both Gran Canaria propolis samples is very similar, while the extracts from the three Tenerife samples show significant differences. For example, in the extract from sample Pr-2, sesquiterpenes, aliphatic alcohols and carbonyl compounds were not detected even at trace levels, while extract Pr-3 did not contain phenolic compounds. These differences may be due to the fact that the Tenerife propolis samples were collected from apiaries located at different altitudinal levels (from 600 to 1400 m above sea level) with different floristic compositions.

A distinctive characteristic of the chemical composition of the studied propolis samples from Tenerife is the presence of a large number and, in two out of three samples, a high content of diterpenoids, mainly resin acids, while in the case of propolis from Gran Canaria, one compound of this class was found with a relative contribution of 0.1% TIC. The most likely plant source of resin acids in propolis from Tenerife is the secretion of an endemic pine species, *Pinus canariensis*. There is no information in the available literature on the chemical composition of the resinous secretions of this species, and our assumption is based on the qualitative composition of diterpene acids in propolis, characteristic of resins of all pine species [34]. The lower boundary of closed pine forests in Tenerife lies at an altitude of 1000–1200 m above sea level, but individual trees are found in plantings right up to the coastline. Thus, the lowest content of resin acids in the extract of sample Pr-3, collected in the San Miguel region at an altitude of about 600 m a.s.l., can be explained by the limited availability of this source of resin for bees.

Another difference concerns the composition of phenolic compounds, represented in Tenerife propolis by alkyl derivatives of resorcinol and salicylic acid, but absent in propolis samples from Gran Canaria. A probable plant source of these lipids is *Mangifera indica* (Anacardiaceae), brought from Indonesia to the Canary Islands at the end of the 18th century and widely cultivated on all the islands of the archipelago. These compounds were first discovered in Indonesian propolis [35] and later in Thai propolis [36]. Both publications name *M. indica* as the source of these compounds. Their absence in sample Pr-3, as well as in propolis samples from Gran Canaria, can be explained by the inaccessibility of mango plants to bees. On the other hand, a similar feature of the chemical composition of propolis from Gran Canaria and Tenerife is the high content of furofuranoid lignans. However, the plant precursor of these compounds, which have beneficial effects on brain function [37,38,39], including protection against Parkinson’s disease, Alzheimer’s disease, stroke, and other neurodegenerative diseases, remains unknown.

Thus, based on our research and earlier results [32], we can preliminarily state that Canarian propolis is significantly different in chemical composition from known types of propolis from the mid-latitude and tropical zones, and its distinctive feature is a high content of furofuran lignans.

## 4. Materials and Methods

### 4.1. Chemicals and Material

Silylation agent, bis(trimethylsilyl)trifluoroacetamide (BSTFA) with addition of 1% of trimethylchlorosilane, was purchased from TriMen Chemicals (Lodz, Poland).

Propolis samples were collected by one of the authors of the article in January, April and August 2023 from hives in different apiaries. Two samples were obtained in the Santiago del Teide area. The first of them (Pr-1) comes from an apiary located at an altitude of 1020 m above sea level (28°8′ N–16°47′ W) and the second (Pr-2) from an apiary located at an altitude of 1440 m above sea level (28°17′ N–16°46′ W). The third sample (Pr-3) was obtained from an apiary in the San Miguel area at an altitude of about 630 m above sea level (28°05′ N–16°37′ W).

### 4.2. Determination of Volatiles

Determination of the volatile components of the propolis was carried out using HS-SPME/GC-MS. Dry propolis (1.0–1.5 g) was frozen at −18 °C, crushed to a particle size of 1–2 mm and placed in a 16 mL HS-SPME vial with a screw cap and a silicone membrane. The membrane was pierced with the needle of a SPME device with DVB/CAR/PDMS fiber. Every 15–20 min the contents of the vial were shaken to mix the gas phase. After 2 h of exposure at room temperature (21 ± 1 °C), the fiber was placed into the injection port of an HP7890A gas chromatograph with a 5975C VL MSD Triple-Axis Detector (Agilent Technologies, Santa Clara, CA, USA) for 15 min. The apparatus was fitted with an HP-5ms capillary column (30 m × 0.25 mm i.d., 0.25 μm film thickness) with electronic pressure control and a split/splitless injector. The latter was operated at 220 °C in splitless mode. The helium flow rate through the column was 1 mL min^−1^. The initial column temperature was 40 °C and rose to 180 °C at a rate of 3 °C min^−1^. The MSD detector acquisition parameters were as follows: the transfer line temperature was 280 °C, the MS source temperature was 230 °C, and the MS quad temperature was 150 °C. The EI mass spectra were obtained at 70 eV of ionization energy. Detection was performed in the full scan mode. After integrating and summing the areas of the recorded peaks, the fraction of separated components in the total ion current (TIC) was calculated. All analyses were carried out in duplicate.

In a separate experiment, the retention times of the *n*-alkanes used as standards in the calculation of chromatographic retention indices on the above-mentioned GC/MS equipment used were determined. From 0.5 to 10 µL of C_5_–C_17_ *n*-alkanes were injected into a 16 mL vial for HS-SPME with a silicone membrane containing 5 mL of pure glycerol using a 10 µL microsyringe, increasing the dose for each subsequent homologue. The mixture was thoroughly mixed, and DVB/CAR/PDMS fiber was introduced into the gas phase above it for 2–3 s. The chromatogram of reference alkanes was recorded under the above conditions.

### 4.3. Determination of Extractive Components

After concentrating the volatile compounds on the sorption fiber for SPME, the propolis was transferred from the vial to a 50 mL conical flask, and 25 mL of diethyl ether was added and stirred on a magnetic stirrer for 30 min. The solvent was separated, and the extraction was repeated twice. The combined extracts were filtered through a paper filter, and the ether was completely removed on a rotary evaporator. Five milligrams of the viscous residue was transferred into a 2 mL vial and dissolved in 220 µL of dry pyridine, 80 µL of BSTFA was added and the resulting solution was heated for 30 min at a temperature of 60 °C.

The resulting TMS derivatives were separated on the above-mentioned GC-MS apparatus, equipped with the same HP-5ms column. The initial temperature of the column thermostat was 50 °C and increased linearly at a rate of 3 °C min^−1^ to 320 °C. The chromatograph injector, heated to 280 °C, operated with a division of the carrier gas flow (1:10). The helium flow rate through the column was 1 mL min^−1^ in constant flow mode.

Under the given conditions, a calibration mixture of C_10_–C_40_ *n*-alkanes was separated, and the recorded retention times were used to calculate *RI^Exp^* values in the chromatograms of the extracts.

### 4.4. Component Identification

To identify the components, mass spectrum and chromatographic retention index (*RI*) were used. The mass spectrometric identification of volatiles was carried out using an automatic system for GC-MS data processing supplied by the NIST 14 library, as well as by computer search libraries containing the spectra and *RI* values from Adams’ [40] and Tkachev’s [41] collections. The *RI* values of TMS derivatives were compared with those in the NIST collection [42], as well as with those presented in a recently published atlas containing mass spectra and retention indices of more than 1750 organic components of various origins in the form of TMS derivatives, including bee products [43]. An identification was considered reliable if the results of the computer search of the mass spectra library were confirmed by the experimental *RI^Exp^* values, i.e., if their deviation from the published database values (*RI^Lit^*) did not exceed ±10 u.i. If the result of mass spectrometric identification was not confirmed chromatographically due to the absence of *RI* values in the available databases, or if the *RI^Exp^* and *RI^Lit^* values differed by more than 10 u.i., the identification was considered tentative (the names of the tentatively identified compounds in Table 1 and Table 2 are followed by a question mark).

## Figures and Tables

**Table 1:** Relative group composition (% TIC) of volatile compounds in Canary (Tenerife) propolis.

Monoterpene/Monoterpenoids, Including:	*RI* *^Exp^*	*RI^Lit^*	Sample		
			Pr-1	Pr-2	Pr-3
			27.07	55.84	38.10
tricyclene	919	921	0.19	0.16	0.21
α-thujene	925	926	1.35	N.d. *	2.58
α-pinene	936	936	6.43	10.07	18.68
camphene	945	946	0.59	1.03	0.28
dehydrosabinene	956	957	N.d.	1.49	N.d.
sabinene	970	973	0.68	N.d.	5.55
β-pinene	975	975	0.42	2.43	1.06
myrcene	990	991	0.36	1.54	0.51
3-carene	1010	1011	0.26	trace **	1.76
α-terpinene	1016	1017	0.18	N.d.	0.42
limonene	1028	1028	13.36	4.77	1.49
*cis-*β-ocimene	1044	1042	N.d.	0.56	N.d.
*trans-*β-ocimene	1050	1048	N.d.	0.21	N.d.
γ-terpinene	1056	1057	0.30	N.d.	0.86
dihydromyrcenol	1071	1073	0.35	N.d.	N.d.
terpinolene	1086	1088	N.d.	N.d.	0.57
*trans*-sabinene hydrate	1096	1097	N.d.	N.d.	0.19
α-campholenal	1124	1226	N.d.	2.18	0.47
*trans-*pinocarveol	1135	1140	0.54	1.97	0.35
*trans-*verbenol	1142	1142	0.51	0.57	0.49
pinocarvone	1161	1164	N.d.	1.22	N.d.
borneol	1165	1168	0.36	1.01	N.d.
isopinocamphone	1174	1175	N.d.	0.29	N.d.
4-terpineol	1178	1178	0.11	0.84	1.16
α-terpineol	1189	1191	0.12	7.82	0.23
α-thujenal	1195	1190	N.d.	0.38	N.d.
myrtenol	1198	1196	N.d.	1.26	N.d.
verbenone	1209	1212	N.d.	2.20	N.d.
*trans-*carveol	1220	1218	N.d.	2.40	N.d.
*cis*-carveol	1230	1229	N.d.	0.59	N.d.
carvone	1243	1245	N.d.	0.92	N.d.
carvotanacetone	1246	1249	N.d.	0.47	N.d.
bornyl acetate	1285	1287	N.d.	3.36	0.30
piperitenone	1340	1340	N.d.	0.20	-
**Sesquiterpene/sesquiterpenois, including:**			**16.50**	**12.18**	**3.78**
δ-elemene	1340	1342	N.d.	N.d.	1.17
α-cubebene	1350	1351	2.43	N.d.	N.d.
α-longipinene	1353	1357	N.d.	0.40	N.d.
α-copaene	1376	1376	0.41	0.16	N.d.
β-bourbonene	1383	1385	N.d.	1.21	N.d.
sativene?	1388	1394	N.d.	0.27	N.d.
β-cubebene	1391	1392	0.31	N.d.	N.d.
β-elemene	1393	1391	0.28	N.d.	N.d.
longifolene	1405	1405	N.d.	2.08	N.d.
acora-3,7(14)-diene	1408	1408	N.d.	0.29	N.d.
β-funebrene	1410	1412	N.d.	N.d.	0.29
β-caryophyllene	1417	1419	3.49	3.29	1.10
β-copaene	1433	1432	N.d.	0.19	N.d.
γ-elemene	1433	1433	N.d.	N.d.	0.23
α-humulene	1454	1454	0.30	1.16	N.d.
γ-muurolene	1478	1479	0.39	0.21	N.d.
β-selinene	1485	1486	0.55	N.d.	N.d.
valencene	1494	1493	0.47	N.d.	N.d.
α-selinene	1496	1496	0.31	N.d.	N.d.
α-muurolene	1503	1500	0.19	N.d.	N.d.
β-bisabolene	1509	1508	N.d.	0.13	N.d.
γ-cadinene	1516	1515	0.41	0.19	N.d.
δ-cadinene	1526	1524	0.96	N.d.	0.14
selina-4(15),7(11)-diene? ***	1536	-	0.21	N.d.	N.d.
selina-3,7(11)-diene	1542	1541	0.25	N.d.	N.d.
elemol	1555	1550	0.45	N.d.	N.d.
germacrene B	1557	1557	N.d.	N.d.	0.49
caryophyllene oxide	1582	1583	0.66	1.39	N.d.
cedrol	1600	1600	0.25	N.d.	0.37
humulene epoxide II	1608	1606	N.d.	0.19	N.d.
eremoligenol	1630	1630	1.71	N.d.	N.d.
caryophylladienol II	1635	1636	N.d.	0.12	N.d.
β-eudesmol	1648	1650	1.15	N.d.	N.d.
α-eudesmol	1651	1653	0.92	N.d.	N.d.
14-hydroxy-β-caryophyllene?	1657	1667	N.d.	0.59	N.d.
unidentified sesquiterpenol C_15_H_22_O_2_	1662	-	0.24	N.d.	N.d.
**Diterpene hydrocarbons, including:**			**0.40**	N.d.	N.d.
unidentified diterpene C_20_H_32_	1762	-	0.15	N.d.	N.d.
unidentified diterpene C_20_H_32_	1805	-	0.25	N.d.	N.d.
**Aliphatic acid, including:**			**7.3**	**1.94**	**6.23**
formic acid	538	535	2.78	trace	1.32
acetic acid	626	616	3.03	1.94	4.64
isobutyric acid	765	762	0.72	trace	N.d.
isovaleric acid	842	848	0.32	N.d.	0.14
2-methylbutanoic acid	862	868	trace	N.d.	0.13
hexanoic acid	983	989	0.27	N.d.	N.d.
**Aliphatic alcohol, including:**			**4.87**	**0.82**	**1.75**
ethanol	480	484	2.64	0.03	0.81
isopentanol	730	734	0.26	trace	0.53
2,3-butanediol, isomer 1	737	734	0.22	N.d.	0.15
3-methyl-2-buten-1-ol (prenol)	770	771	0.81	N.d.	N.d.
2,3-butanediol, isomer2	782	779	0.52	N.d.	0.35
1-hexanol	866	870	0.44	N.d.	N.d.
1-heptanol	970	968	N.d.	0.13	N.d.
1-octanol	1070	1070	N.d.	0.40	N.d.
**Esters, including:**			**2.96**	N.d.	N.d.
*n*-propyl propionate	810	808	0.16	N.d.	N.d.
*n-*butyl butanoate	997	998	0.32	N.d.	N.d.
*n-*butyl hexanoate	1193	1192	1.68	N.d.	N.d.
ethyl octanoate	1199	1198	0.17	N.d.	N.d.
glycerol 1,2-diacetate?	1355	-	0.41	N.d.	N.d.
*n-*hexyl hexanoate	1387	1387	0.22	N.d.	N.d.
**Aliphatic carbonyls, including:**			**20.13**	**12.76**	**8.90**
acetone	500	501	2.73	2.31	2.28
isopentanal	648	648	trace	N.d.	N.d.
Acetol (hydroxyacetone)	667	673	N.d.	N.d.	0.48
acetoin (3-hydroxy-butanone)	722	722	0.51	N.d.	1.00
3-methyl-2-butenal (prenal)	780	776	0.79	0.34	0.26
hexanal	801	801	1.97	071	N.d.
2-hexenal	851	853	N.d.	0.36	N.d.
3-heptanone	885	890	N.d.	N.d.	0.22
heptanal	902	902	1.12	0.80	0.19
*trans*-2-heptenal	954	956	N.d.	0.13	N.d.
6-methyl-5-hepten-2-one	986	987	0.28	0.24	0.66
octanal	1002	1004	0.52	1.68	1.42
2-nonanone	1092	1089	N.d.	0.95	N.d.
nonanal	1103	1104	6.34	3.17	1.91
2,6-(*E*,*Z*)-nonadienal	1153	1156	N.d.	0.38	N.d.
decanal	1208	1207	2.11	0.63	N.d.
(*E*)-2-decenal	1261	1261	N.d.	0.40	N.d.
2-undecanone	1296	1294	N.d.	0.66	N.d.
undecanal	1309	1308	0.02	N.d.	N.d.
**Aromatics, including:**			**7.38**	**3.99**	**6.61**
toluene	761	761	2.80	0.37	1.03
*p*-xylene	865	866	N.d.	N.d.	0.16
styrene	894	893	N.d.	2.65	N.d.
benzaldehyde	964	960	0.11	0.46	N.d.
*p*-cymene	1022	1023	4.44	0.52	2.14
*m-*cymen-8-ol	1181	1184	0.02	N.d.	N.d.
*p-*cymen-8-ol	1184	1187	0.14	N.d.	0.28
**Alkane & alkene, including:**			**6.00**	**9.33**	**32.48**
*n*-hexane	600	600	4.30	N.d.	1.50
*n*-heptane	700	700	1.39	0.18	0.68
1-octene	790	791	0.31	N.d.	0.30
*n*-octane	800	800	N.d.	N.d.	0.54
*n-*nonane	900	900	trace	trace	0.12
2-methylnonane	962	962	N.d.	N.d.	0.13
*n-*decane	1000	1000	N.d.	trace	6.03
4-methyldecane	1060	1060	N.d.	0.40	0.25
2-methyldecane	1063	1063	N.d.	0.18	1.55
3-methyldecane	1070	1070	N.d.	N.d.	0.48
*n-*undecane	1100	1100	N.d.	0.45	2.05
2,6-dimethyldecane	1109	1109	N.d.	0.63	N.d.
2,9-dimethyldecane	1127	1126	N.d.	N.d.	0.13
6-methylundecane	1163	1062	N.d.	N.d.	0.61
1-dodecene	1190	1193	N.d.	N.d.	0.22
*n*-dodecane	1200	1200	N.d.	0.98	11.85
4-methyldodecane	1260	1259	N.d.	N.d.	0.15
2-methyldodecane	1263	1263	N.d.	N.d.	0.77
*n-*tridecane	1300	1300	N.d.	N.d.	0.78
*n-*tetradecane	1400	1400	N.d.	trace	2.28
*n-*pentadecane	1500	150	N.d.	1.64	0.32
*n*-heptadecane	1600	1600	N.d.	N.d.	0.50
**Other, including:**			**4.25**	**1.16**	**1.47**
chloroform	615	615	3.67	N.d.	N.d.
pyridine	742	742	N.d.	N.d.	1.10
furfural	830	834	0.33	0.17	N.d.
γ-butyrolactone	916	914	N.d.	0.14	N.d.
3,7,7-trimethyl-1,3,5-cycloheptatriene	967	970	0.25	N.d.	0.37
γ-caprolactone	1056	1060	N.d.	0.39	N.d.
4-acetyl-1-methylcyclohexene	1130	1131	N.d.	0.25	N.d.
2-methylene-6,6-dimethylbicyclo [3.2.0]heptan-3ol	1156	1157	N.d.	0.21	N.d.
**NN**			**4.55**	**1.98**	**2.68**

* N.d.—Not detected; ** trace—below 0.01% TIC; *** ?—tentatively.

**Table 2:** Relative group composition (% TIC) of ether extracts of Canarian propolis.

Group of Compounds	*RI* *^Exp^*	*RI^Lit^*	Sample		
			Pr-1	Pr-2	Pr-3
**Sesquiterpene/Sesquiterpenoids, Including:**			**1.71**	N.d. *	**1.28**
β-caryophyllene	1417	1419	0.05	N.d.	0.07
caryophyllene oxide	1583	1583	0.07	N.d.	0.06
α-copaen-11-ol, TMS	1634	1636	0.13	N.d.	N.d.
elemol, TMS	1637	1638	0.06	N.d.	N.d.
τ-cadinol, TMS	1699	1701	0.05	N.d.	N.d.
α-acorenol, TMS	1723	1722	0.14	N.d.	0.05
agarospirol, TMS	1734	1734	0.05	N.d.	N.d.
γ-eudesmol, TMS	1744	1741	0.76	N.d.	0.38
β-eudesmol, TMS	1753	1750	0.40	N.d.	0.18
(2*E*,6*Z*)-farnesol, TMS	1814	1811	0.05	N.d.	N.d.
**Diterpenoids, including:**			**10.42**	**55.18**	**2.48**
pimaric acid, TMS	2301	2302	0.60	5.47	N.d.
sandaracopimaric acid, TMS	2319	2318	0.42	1.45	N.d.
*trans*-communic acid, TMS	2325	2324	0.17	trace **	0.11
isopimaric acid, TMS	2332	2333	1.39	7.68	0.39
totarol, TMS? ***	2338	3332	0.35	N.d.	N.d.
palustric acid, TMS	2360	3357	N.d.	4.78	N.d.
diterpene aldehyde C_20_H_30_O (MW 286)	2367	-	N.d.	0.40	N.d.
communic acid, TMS	2377	3375	N.d.	3.58	N.d.
dehydroabietic acid, TMS	2388	2386	1.76	6.93	0.20
abietic acid, TMS	2414	2414	0.89	8.44	0.14
13-*epi*-cupressic acid, di-TMS	2438	2435	0.63	N.d.	0.16
podocarpic acid, di-TMS?	2482	N.d.	0.17	N.d.	N.d.
neoabietic acid, TMS	2508	2508	N.d.	3.55	N.d.
unidentified diterpenoid, TMS	2515	N.d.	N.d.	1.31	N.d.
15-hydroxydehydroabietic acid, di-TMS	2540	2536	0.61	1.74	N.d.
imbricatoloic acid, di-TMS	2550	2548	0.29	1.19	N.d.
unidentified diterpenoid, TMS	2665	-	N.d.	1.51	N.d.
isocupressic acid, di-TMS	2592	2592	N.d.	0.86	1.48
unidentified diterpenoid, TMS	2596	-	1.65	3.60	N.d.
pinifolic acid, di-TMS	2640	2644	0.82	N.d.	N.d.
7a,15-dihydroxydehydroabietic acid, tri-TMS	2748	2744	0.09	0.61	N.d.
15-hydroxy-7-oxodehydroabietic acid, di-TMS	2787	2789	0.08	N.d.	N.d.
**Triterpenoids, including:**			**21.4**	**7.48**	**5.31**
unidentified triterpenoid, TMS (393,73,149,69)	3265	-	0.61	N.d.	N.d.
dihydrolanostreol, TMS?	3292	-	0.17	N.d.	N.d.
β-amyrone?	3307	-	0.53	0.12	N.d.
lanosterol, TMS	3332	3335	0.25	trace	0.30
β-amyrin, TMS	3350	3347	0.72	1.40	N.d.
olean-18-en-3-ol ? TMS	3360	-	1.23	0.62	0.3
α-amyrin, TMS	3380	3378	0.05	2.48	N.d.
lupeol, TMS	3395	3401	N.d.	0.88	0.20
cycloartenol? TMS	3407	-	1.16	0.75	N.d.
9,19-cyclolanostan-3-ol, 24-methylene-? TMS	3468	-	0.68	0.16	N.d.
-masticadienoic acid? TMS	3702	-	2.29	N.d.	1.04
unidentified triterpenoid, TMS (95,511,189,526)	3776	-	1.79	N.d.	N.d.
unidentified triterpenoid, TMS	3807	-	1.79	N.d.	0.52
**Resorcinol derivatives, including:**			**1.17**	**0.54**	**N.d.**
(Z,Z)-5-heptadec-9,12-dienylresorcinol, di-TMS	2877	2881	0.54	0.41	N.d.
(5Z)-5-heptadecenylresorcinol, di-TMS	2903	2905	0.23	trace	N.d.
5-heptadecylresorcynol, di-TMS	2908	2911	0.05	trace	N.d.
5-nonadecenylresorcynol, di-TMS	3101	3102	0.35	0.13	N.d.
**Salicylic acid derivatives, including:**			**1.22**	N.d.	N.d.
ginkgolic acid, C15:1, di-TMS	2861	2862	0.13	N.d.	N.d.
salicylic acid, 6-heptadecadienyl-, di-TMS	3031	3026	0.10	N.d.	N.d.
ginkgolic acid, C17:1, di-TMS	3059	3056	0.27	N.d.	N.d.
salicylic acid, 6-heptadecyl-, di-TMS	3063	3061	0.11	N.d.	N.d.
salicylic acid, 6-(12-hydroxyheptadecyl)-, tri-TMS	3260	3261	0.61	N.d.	N.d.
**Lignans, including:**			**21.29**	**3.30**	**6.65**
(+)-epi-sesamin	3140	3140	2.27	0.35	0.73
fargesin	3201	3202	3.03	0.64	1.02
eudesmin? (pinoresinol, dimethyl ether)	3250	-	0.76	0.22	0.35
aschantin	3333	3332	4.84	1.51	1.82
magnolin	3369	3371	0.95	0.12	0.28
(+)-magnolin	3388	3388	2.28	N.d.	0.76
yangambin, isomer 1	3510	3510	1.44	0.27	0.29
yangambin, isomer 2	3515	3519	2.18	0.69	1.41
unidentified lignan (430,179,165,181,207)	3568	-	2.58	0.50	N.d.
**Aliphatic acids, including:**			**15.85**	**10.99**	**21.18**
azelaic acid, di-TMS	1807	1806	0.13	0.14	0.05
hexadecanoic acid, TMS	2052	2051	1.92	1.53	1.90
linoleic acid, TMS	2215	2215	0.22	0.60	0.26
oleic acid, TMS	2222	2222	2.18	4.14	2.66
(*E*)-vaccenic acid, TMS	2229	2233	0.09	N.d.	N.d.
octadecanoic acid, TMS	2249	2250	0.58	0.16	0.40
(*Z*)-11-eicosenoic acid	2420	2419	0.07	N.d.	N.d.
3-hydroxyoctadecanoic acid, di-TMS	2429	2429	0.07	N.d.	N.d.
eicosanoic acid, TMS	2448	2447	0.12	N.d.	0.15
heneicosanoic acid, TMS	2548	2546	0.18	N.d.	N.d.
docosanoic acid, TMS	2644	2645	0.72	N.d.	1.50
tricosanoic acid, TMS	2743	2747	0.23	N.d.	0.18
tetracosanoic acid, TMS	2847	2845	3.08	2.84	5.96
hexacosanoic acid, TMS	3044	3043	1.43	0.24	2.12
23-hydroxytetradecanoic acid, di-TMS	3115	3118	0.22	0.11	0.24
octacosanoic acid, TMS	3242	3241	1.24	trace	1.90
triacontenoic acid, TMS	3422	-	N.d.	N.d.	0.22
triacontanoic acid, TMS	3442	3440	1.68	N.d.	1.31
dotriacontenoic acid, TMS	3623	-	N.d.	N.d.	0.20
dotriacontanoic acid, TMS	3642	3641	0.60	N.d.	0.58
tetratriacontanoic acid, TMS	3838	3838	0.86	N.d.	0.61
**Aliphatic alcohols, including:**			**1.01**	**N.d.**	**2.99**
1-octadecanol, TMS	2164	2165	0.09	N.d.	N.d.
1-tetracosanol, TMS	2753	2754	0.43	N.d.	0.26
1-hexacosanol, TMS	2949	2951	0.22	N.d.	0.20
1-octacosanol, TMS	3148	3148	0.24	N.d.	0.36
1-triacontanol, TMS	3346	3346	N.d.	N.d.	1.13
1-dotriacontanol, TMS	3546	3542	N.d.	N.d.	0.66
1-tetratriacontanol, TMS	3742	3741	N.d.	N.d.	0.26
**Aliphatic carbonyls, including:**			**0.43**	**N.d.**	**N.d.**
tricosanal	2533	2534	0.36	N.d.	N.d.
hexacosanal	2835	2833	0.07	N.d.	N.d.
**Alkane & alkenes, including:**			**14.94**	**28.20**	**47.59**
*n*-heptadecane	1700	1700	N.d.	N.d.	0.09
*n*-nonadecane	1900	1900	0.14	trace	0.37
*n*-eicosane	2000	2000	N.d.	N.d.	0.07
*n*-heneicosane	2100	2100	0.26	1.37	0.80
9-(*Z*)-tricosene	2270	2271	N.d.	N.d.	0.29
*n*-tricosane	2300	2300	1.01	6.87	2.34
*n*-tetracosane	2400	2400	0.17	N.d.	0.35
9-pentacosene	2473	2475	0.09	0.51	0.35
7-pentacosene	2478	2482	N.d.	N.d.	0.09
*n*-pentacosane	2500	2500	1.63	2.87	4.01
*n*-hexacosane	2600	2600	0.36	N.d.	1.48
*n*-heptacosane	2700	2700	3.94	0.70	8.37
13-methylheptacosane	2731	2731	0.23	N.d.	0.40
*n*-octacosane	2800	2800	0.27	N.d.	0.58
2-methyloctacosane	2860	2858	N.d.	N.d.	0.15
9-nonacosene	2876	2875	N.d.	N.d.	1.87
7-nonacosene	2885	2882	N.d.	2.63	N.d.
*n*-nonacosane	2900	2900	2.67	0.70	6.72
9-triacontene	2983	2984	N.d.	N.d.	0.33
*n*-triacontane	3000	3000	0.19	N.d.	0.36
9-hentriacontene	3073	3075	0.48	3.44	2.75
7-hentriacontene	3081	3082	0.89	4.21	3.39
*n*-hentriacontane	3100	3100	1.42	N.d.	4.09
9-tritriacontane	3276	3277	0.82	4.24	5.49
7-tritriacontane	3282	3282	0.26	N.d.	N.d.
*n*-tritriacontane	3300	3300	0.31	N.d.	0.56
**Other, including:**			**4.10**	**5.48**	**0.14**
glycerol, tri-TMS	1293	1294	trace	2.11	0.14
3,4,5-trimethoxybenzoic acid, TMS	1832	1833	0.18	N.d.	N.d.
quinic acid, penta-TMS	1900	1900	N.d.	0.61	N.d.
1-*O*-octadecyl glycerol, di-TMS	2699	2695	N.d.	2.09	N.d.
1-*O-*eicosyl glycerol, di-TMS	2892	2893	1.01	0.67	N.d.
tetracosyl hexadecanoate	>4000	-	2.91	N.d.	N.d.
**NN**			**6.84**	**10.50**	**3.49**

* N.d.—Not detected; ** trace—below 0.01% TIC; *** ?—tentatively.

## Data Availability

The study did not report any data.
